# Interleukin-1 antagonists in Mevalonate Kinase Deficiency

**DOI:** 10.1186/1546-0096-9-S1-P7

**Published:** 2011-09-14

**Authors:** C Galeotti, L Rossi-Semerano, S Guillaume, A Duquesne, P Pillet, O Richer, E Hachulla, I Koné-Paut

**Affiliations:** 1 Department of Pediatric Rheumatology, CEREMAI, CHU Bicêtre, university of Paris Sud, France; 2 Department of Pediatric Rheumatology, CHU Lyon, France; 3 Department of Pediatric Rheumatology, CHU Bordeaux, France; 4 Department of Internal Medicine, CHRU Lille, France

## Background

Mevalonate Kinase Deficiency (MKD) is one of the autoinflammatory fever syndromes, caused by mutations in the *MKD* gene. Systemic inflammatory symptoms may be mild to severe leading to early death, and recurrent bacterial infections frequently develop in the disease course. On demand NSAID and steroids are the most commonly used. Few case-reports suggested that interleukin-1 (IL-1) antagonists could alleviate MKD symptoms.

## Aim

We describe efficacy and safety of IL-1 targeting drugs, anakinra and canakinumab, in seven children with MKD.

## Methods

A questionnaire containing items of disease activity was sent to French pediatric and adult rheumatologists. We collected information from 7 MKD patients treated with IL-1 antagonists. MKD was suspected clinically then confirmed genetically in all cases.

## Results

The use of IL-1 antagonists was beneficial to all patients on a long term. Anakinra was daily administered and canakinumab every 8 weeks, with dose adjustments. The median clinical score based on fever and symptoms during MKD crisis before and after treatment was 9 and 0,5 respectively. The number of days of fever during crisis before and after treatment was 5,25 and 1,75 respectively. These treatments alleviated biological inflammatory markers. The side effects with anakinra were local pain, inflammatory signs at the injection site, infections (bacterial pneumopathy and rhinopharyngitis) and acne. There was no side effect with canakinumab. Three patients switched from anakinra to canakinumab.

## Conclusion

IL-1 targeting drugs appear effective and safe in our MKD patients. Controlled trials to further assess their clinical benefit and safety in MKD patients seem necessary.

